# Identification of DLEC1 D215N Somatic Mutation in Formalin Fixed Paraffin Embedded Melanoma and Melanocytic Nevi Specimens

**DOI:** 10.1155/2013/469671

**Published:** 2013-10-13

**Authors:** Ricardo Vieira, Maria José Simões, Susana Carmona, Conceição Egas, Carlos Faro, Américo Figueiredo

**Affiliations:** ^1^Serviço de Dermatologia, Centro Hospitalar e Universitário de Coimbra, Praceta Mota Pinto, 3000-375 Coimbra, Portugal; ^2^Unidade de Serviços Avançados, Biocant, Parque Tecnológico de Cantanhede, Núcleo 04, Lote 3, 3060-197 Cantanhede, Portugal

## Abstract

DLEC1 has been suggested as a tumor suppressor gene in several cancers. DLEC1 D215N somatic mutation (COSM36702) was identified in a melanoma cell line through whole genome sequencing. However, little is known about the implication and prevalence of this mutation in primary melanomas or in melanocytic nevi. The aim of this study was to genotype DLEC1 D215N mutation in melanoma tissue and melanocytic nevi samples to confirm its occurrence and to estimate its prevalence. Primary melanomas (*n* = 81) paired with synchronous or asynchronous metastases (*n* = 21) from 81 melanoma patients and melanocytic nevi (*n* = 28) were screened for DLEC1 D215N mutation. We found the mutation in 3 primary melanomas and in 2 melanocytic nevi, corresponding to a relatively low prevalence (3.7% and 7.1%, resp.). The pathogenic role of DLEC1 215N mutation is unclear. However, since the mutation has not been previously described in general population, its involvement in nevogenesis and melanoma progression remains a possibility to be clarified in future studies.

## 1. Introduction

Mutations in deleted in lung and esophageal cancer 1 (DLEC1) gene or its inactivation by epigenetic silencing, namely, promoter CpG island hypermethylation or histone hypoacetylation, were previously reported in several cancers (lung [1], esophagus [2], kidney [3], stomach [4], colon [4], ovary [5], breast [6], head and neck [7], and lymphoma [8]). Furthermore, a negative impact on the prognosis related with DLEC1 inactivation was demonstrated in lung [9], kidney [10], and ovary [5] carcinomas.

DLEC1 D215N mutation (COSM36702) is a G > A substitution in codon 641 (ENST00000308059) which was identified in whole genome sequencing of a tumor cell line derived from melanoma metastases [11]. To establish the catalogue of somatic mutations in cancer cells, a lymphoblastoid line derived from the same patient was also sequenced [11]. Three different missense mutations were found in other genome or exome sequencing studies, making DLEC1 a candidate tumor suppressor gene in cutaneous melanoma, probably acting by inhibition of cell proliferation [12, 13]. Nevertheless, the prevalence of DLEC1 mutations among primary melanomas, melanoma metastases, or benign melanocytic nevi remains undetermined.

The aim of this study was to confirm the occurrence and estimate the prevalence of DLEC1 D215N mutation in formalin fixed and paraffin embedded tissue samples from melanoma and melanocytic nevi.

## 2. Patients and Methods

### 2.1. Melanoma Patients

In all, 102 formalin fixed paraffin embedded tumor tissues from 81 patients with melanoma were screened. Samples of primary melanomas (*n* = 81) paired with synchronous or asynchronous skin metastases (*n* = 20) or lymph node metastases (*n* = 1) from the same patients underwent genotyping assay for DLEC1 D215N mutation. The 81 patients were selected from a set of 224 patients treated for primary cutaneous melanoma at the Department of Dermatology of Coimbra University Hospital, Portugal, between 2000 and 2008. The exclusion of 143 out of 224 patients was based on the following criteria: (i) patient loss from followup (*n* = 16), (ii) no primary tumor samples available for screening due to primary melanoma removal performed in other centers (*n* = 97) or primary unknown melanoma (*n* = 3), (iii) DNA isolation from the available samples of the patient did not achieve DNA concentrations or absorbance ratios required for genotyping analysis (*n* = 19 : 24 samples), and (iv) the genotyping assay for DLEC1 mutation was not successful in patient's samples (*n* = 8, 9 samples). The clinical data of melanoma patients are disclosed on Table 1.

### 2.2. Melanocytic Nevus Patients

Simultaneously, 28 formalin fixed paraffin embedded tissue from acquired benign melanocytic nevi (without atypical features) from 28 patients were also screened. The 28 patients were selected from a group of 70 patients submitted to melanocytic nevus surgical excision during a 6-month period in 2008. Cosmetic concern was the main reason for nevus excision. Forty-two out of 70 patients were excluded due to (i) patient with no nevus sample available for screening (*n* = 4), (ii) DNA isolation from the available samples of the patient did not achieve DNA concentrations or absorbance ratios required for genotyping (*n* = 32), and (iii) the genotyping assay for DLEC1 mutation was not successful in patient's sample (*n* = 6). The clinical data of melanocytic nevi patients are disclosed in Table 2.

### 2.3. Preparation and Quality Control of DNA Samples

Formalin fixation and paraffin embedding effectively preserve tissue morphological details, allowing easy storage at room temperature for long periods. However, this preservation method impairs DNA extraction efficacy and quality, limiting the molecular analyses and affecting the genotyping results [14]. There is evidence for the influence of fixation and embedding procedures on the nucleic acids fragmentation and appearance of artifactual mutations or false-negatives [15]. These artifacts should be prevented by using large amounts of double-stranded DNA, by performing multiple amplifications or by using appropriated DNA FFPE tissue extraction kits which partially reverse formalin modifications of nucleic acids. 

Given the high heterogeneity of the tissues, composed by heterogeneous populations of normal and tumor cells, false-negative results due to amplification of normal cells are also problematic in this kind of analysis. Thus, it is strongly recommended to include only specimens with more than 50% of tumor cells. This recommendation was followed when the samples were harvested from paraffin blocks, cutting off the remaining normal tissue for enhancing tumor cell representation. 

At least ten 6 *μ*m thickness slices were cut from each tissue paraffin block. DNA samples were isolated from those slices by use of the QIAamp DNA FFPE Tissue Kit (QIAGEN, Hilden, German). Quality control of the isolated DNA was performed by measuring both 260/230 nm and 260/280 nm absorbance ratios on a NanoDrop spectrophotometer. All samples were quantified by fluorimetry using PicoGreen dsDNA Quantitation Reagent (Molecular Probes, Inc., Eugene, Oregon, US). The recommended starting concentration for genotyping using TaqMan assays (Life Technologies, Carlsbad, California, US) is 50 ng/*μ*L. However, as expected, the majority of the samples were below this value. Upon preliminary tests using lower initial concentrations, we observed that concentrations ranging from 15 to 20 ng/*μ*L also yielded good genotyping results. Whenever possible, DNA samples at initial concentrations lower than 15 ng/*μ*L and/or absorbance ratios below 1.7 were further concentrated and/or purified by precipitation with glycogen and isopropanol. Only samples with at least 15 ng/*μ*L and absorbance ratios of 1.6 or greater were considered for genotyping analysis.

### 2.4. Genotyping Analysis

DNA samples were genotyped for DLEC1 D215N mutation using the TaqMan OpenArray Genotyping System (Life Technologies, Carlsbad, CA, US) at the Genoinseq, BIOCANT-Biotechnology Innovation Center, Cantanhede, Portugal. Given the nature of these DNA samples, namely, the high heterogeneity of the tissues, each specimen was genotyped at least three times. This DLEC1 D215N genotyping assay was included in a TaqMan OpenArray plate. DNA samples were normalized to 15 ng/*μ*L and a total of 45 ng was used for genotyping of the mutation. Two nontemplate controls were used to determine the genotyping clusters and check for contaminations. Before plate loading, TaqMan OpenArray Master Mix was added to the normalized DNA samples (1 : 1). Sample loading into the plates was done by using the OpenArray Accuffil Instrument (Life Technologies, Carlsbad, CA, US). Thermal cycling was performed in a Dual Flat Block GeneAmp PCR System 9700 (Life Technologies, Carlsbad, California, US), according to the manufacturer's protocol. Plate imaging, that is, the acquisition of genotypes, was done by use of the OpenArray NT Imager with the OpenArray SNP Genotyping Analysis Software (Life Technologies, Carlsbad, CA, US). During imaging, the OpenArray NT Imager recorded the amount of fluorescence in each through-hole of the plates. Genotyping data analysis was performed using the TaqMan Genotyper Software (Life Technologies, Carlsbad, California, US) by autocalling as the call method. All calls or genotypes were then manually reviewed and corrected if needed.

To determine the clinical significance of the mutation, the 1000 Genome data, representing the common frequency of these mutations in Europe, was used as control. SIFT software was also used to estimate the impact of the mutation on protein function [16].

### 2.5. Statistics

All statistical analyses (chi-square test and Fisher's exact test) were performed using the software IBM SPSS 19. Significance was set a *P* value under 0.05. 

## 3. Results

The samples which do not fulfill the initial requisites of DNA concentration and/or absorbance ratios were excluded, leading to an exclusion rate of 17.8% (24 out of 135) in melanoma tissue and 48.5% (32 out of 66) in melanocytic nevi. This difference was statistically significant (*P* < 0,001).

DLEC1 D215N genotyping call rates were 91.9% (102 out of 111) in melanoma samples and 82.4% (28 out of 34) in melanocytic nevi samples (*P* = 0,119). Therefore, a total of 81 primary melanomas paired with 21 metastases and 28 benign melanocytic nevi samples were successfully genotyped for DLEC1 D215 somatic mutation.

Three primary cutaneous melanomas (3.7%) were mutated in the heterozygous state for DLEC D215N: two cases of superficial spreading melanoma of lower limbs and a case of nodular melanoma of the face, all occurring in female patients. Only the patient with nodular melanoma developed regional lymph node metastases and distant metastases. Nevertheless, no tissue samples of the metastatic disease of that patient were available for mutation screening. DLEC1 D215N heterozygous somatic mutation was also found in two cases (7.1%) of acquired melanocytic nevi: a dermal nevus of the face and a compound nevus in the trunk without atypical features. No statistical significant difference was observed in the prevalence of DLEC D215N mutation between melanoma and melanocytic nevi patients (*P* = 0.6).

The SIFT score of the reported amino acid change (D215N) was 0.33, indicating a tolerated change, probably with low impact on protein function. According to 1000 Genomes data, DLEC D215N mutation was not previously described on general European population.

## 4. Discussion

In spite of the source of the biological material and the limitations in terms of DNA concentrations and absorbance ratios, final genotyping results for DLEC1 D215N assay were very promising, as shown by the genotyping call rates obtained from both melanoma and melanocytic nevi samples. These results suggest the applicability of the TaqMan genotyping technology to study formalin fixed and paraffin embedded tissues. As the amount of tissue influences the efficacy of DNA isolation, the smaller size of melanocytic nevi sample was probably related to the significantly higher failure of DNA isolation compared with melanoma samples (48.5% versus 17.8%).

The majority of melanomas studied were nodular type, contrarily to the expected predominance of superficial spreading type. The overvaluation of nodular melanomas was probably a selection bias caused by referral of high-risk cases from other institutions. The influence of this bias on the prevalence of the mutation was assumed to be low, but its real impact cannot be specifically determined. 

The mutation DLEC1 D215N was detected in a small amount of our samples with no significant difference between melanoma and melanocytic nevi patients. This mutation was described as a somatic mutation in a melanoma cell line [11], but no evidence of a role of this gene in melanoma has been reported and we found no previous data on the occurrence of this mutation in melanoma patients or in melanocytic nevi. Despite the low prevalence and the tolerable impact of the mutation on protein function, as estimated by SIFT software, its occurrence has to be emphasized, since this nucleotide substitution was not observed in European population (1000 Genomes Project). Thus, the likelihood of DLEC D215N mutation being associated with pathogenic events rises sharply, suggesting a potential involvement in nevogenesis and in melanoma development. 

Several oncogene or tumor suppressor gene mutations in melanoma can also be found in melanocytic nevi [17]. In fact, it is not completely elucidated how BRAF or NRAS mutations contribute to the emergence of benign melanocytic nevi as they are also the most frequent mutated genes in melanoma, occurring in early stages of tumor progression [18]. Mechanisms of oncogene-induced cellular senescence were suggested to explain it [19], but recent studies were not able to find senescence traits in human melanocytic nevi [20]. Tumor suppressor genes act in a different way for cancer development. Normally, malignant transformation is preceded by loss of heterozygosity or dominant negative mutations [21]. A loss of function of DLEC1 was previously related with several cancers, suggesting that DLEC1 acts as a tumor suppressor gene. Since the pathogenic role of DLEC1 D215N mutation was not completely elucidated until now, it is not clear if this point mutation contributes with an increased susceptibility for melanoma or for melanocytic nevus development. Consequently, the significance of its occurrence in primary melanomas and in melanocytic nevi remains unclear, claiming for further studies. As well, the association of this mutation with aggressiveness of melanoma needs to be clarified in the future.

## 5. Conclusion

D215N somatic mutation in DLEC1 occurs with a relatively low prevalence in melanocytic nevi and primary melanomas. The detection of this mutation can be effectively performed in formalin fixed paraffin embedded tissues. The potential role of mutated DLEC1 in nevogenesis and melanoma pathogenesis makes DLEC1 a candidate tumor suppressor gene in cutaneous melanoma. Up to our knowledge, this is the first study on prevalence of DLEC1 D215N mutation in melanoma patients and in melanocytic nevi tissue samples.

## Figures and Tables

**Table 1:** Clinical data of melanoma patients.

	Melanoma patients
Mean age (years)	62.6
Gender	
Male	34 (42%)
Female	47 (58%)
Anatomic site	
Head and neck	16 (19.8%)
Trunk	16 (19.8%)
Upper limb	9 (11.1%)
Lower limb	38 (46.9%)
Type	
Lentigo maligna	4 (4.9%)
Superficial spreading	21 (25.9%)
Nodular	31 (38.3%)
Acrolentiginous	22 (27.2%)
Other	3 (3.7%)

**Table 2:** Clinical data of melanocytic nevi patients.

	Melanocytic nevi patients
Mean age (years)	33.2
Gender	
Male	9 (32.1%)
Female	19 (67.9%)
Anatomic site	
Head and neck	10 (35.7%)
Trunk	10 (35.7%)
Upper limb	4 (14.3%)
Lower limb	4 (14.3%)
Type	
Junctional	2 (7.1%)
Dermal	18 (64.3%)
Compound	7 (25%)
Other	1 (3.6%)
